# CT-based optimization of hindlimb muscle measurement levels and development of a hindlimb muscle condition score in dogs

**DOI:** 10.3389/fvets.2026.1854592

**Published:** 2026-07-30

**Authors:** Sukchan Yun, Seri Hong, Yeonjae Lee, Kija Lee, Sooyoung Choi, Daji Noh

**Affiliations:** 1 Departments of Veterinary Medical Imaging, Veterinary Medical Teaching Hospital, Kangwon National University, Chuncheon, Republic of Korea; 2 Departments of Veterinary Medical Imaging, Veterinary Medical Teaching Hospital, Kyungpook National University, Daegu, Republic of Korea

**Keywords:** body composition, computed tomography, cross-sectional area, dog, hindlimb, muscle condition score, skeletal muscle

## Abstract

**Introduction:**

Objective assessment of hindlimb skeletal muscles in dogs remains challenging in small animal practice. This study aimed to use computed tomography (CT) to characterize hindlimb muscle distribution, identify optimal measurement levels, and evaluate the clinical applicability of a palpation-based hindlimb muscle condition score (hMCS) system.

**Methods:**

This prospective observational study included 39 dogs (76 hindlimbs) enrolled according to the predefined inclusion criteria. CT was used to measure the muscle cross-sectional area (CSA) and calculate the muscle ratio (muscle CSA/total CSA) at the three femoral levels. For the tibia, the location of the maximal gastrocnemius muscle CSA was identified, and measurements were performed at that level. Associations with clinical variables [weight, age, sex, body condition score (BCS), hMCS] were analyzed, and the interobserver reliability of the hMCS was evaluated.

**Results:**

Muscle CSA and muscle ratio differed significantly among the femoral levels, with the highest values at the mid-femoral level. In the tibia, the maximal CSA of the gastrocnemius muscle was consistently located in the proximal region. Body weight was positively associated with muscle CSA across all levels, whereas age and BCS were negatively associated with the muscle ratio at most levels. The hMCS showed excellent interobserver reliability and significant associations with CT-derived muscle measurements.

**Discussion:**

The mid-femoral level appears to be a representative site for evaluating femoral muscle mass in dogs, whereas the proximal tibial level provides a practical approach for assessing the crus musculature. Muscle CSA primarily reflects body size, whereas muscle ratio more sensitively reflects compositional changes. The hMCS may serve as a practical complementary tool for hindlimb muscle assessment in dogs.

## 1 Introduction

Skeletal muscles play a fundamental role in regulating movement and contribute to multiple physiological processes, including energy expenditure, maintenance of glucose, lipid, and amino acid homeostasis, modulation of inflammatory responses, and immune function (1, 2). In dogs, the pelvic limb musculature, particularly the thigh and crus, directly contributes to locomotion and postural stability and is frequently considered a functional indicator in clinical assessments (3, 4). Muscle atrophy of the thigh and crus may result from disuse secondary to pain or discomfort, orthopedic and neurological disorders, aging, and chronic systemic diseases, such as congestive heart failure, chronic kidney disease, and neoplasia (5–8). Such muscle loss is associated with reduced strength, impaired immune function, delayed wound healing, decreased quality of life, and potentially poor prognosis (5–9). Furthermore, the evaluation of changes in thigh and crus muscle mass before and after orthopedic procedures, fracture repair, and rehabilitation therapy is considered clinically relevant for assessing functional recovery (10–12).

The muscle condition score (MCS) is a widely adopted clinical scoring system in veterinary clinical practice designed to assess muscle loss primarily through palpation of the epaxial musculature to reflect the overall muscle status (13, 14). Its simplicity, noninvasiveness, and practicality have facilitated its broad clinical use. However, because the conventional MCS primarily reflects generalized muscle conditions, it may not adequately capture region-specific changes in functionally important muscle groups, such as those of the thigh and crus. Therefore, in clinical practice, simple morphometric measurements, such as limb circumference obtained using a measuring tape, have also been used as practical indicators of muscle condition in the pelvic limbs (15, 16). However, thigh musculature may exhibit variations in muscle distribution and muscle cross-sectional area (CSA) along the length of the femur. Standardized anatomical reference levels for clinical or research-based evaluations have not yet been sufficiently established (17, 18). This lack of standardization may limit comparability across studies and affect repeatability within individuals. The gastrocnemius muscle, a major component of the caudal crus musculature, plays a critical role in pelvic limb propulsion and contributes substantially to the overall crus muscle mass (4, 19, 20). Despite its functional importance, independent and systematic quantification of the crus musculature remains relatively limited in the veterinary literature. These considerations highlight the need for more objective and standardized methods for region-specific assessment of thigh and crus musculature.

In human medicine, skeletal muscle mass is commonly quantified using imaging modalities, such as computed tomography (CT), magnetic resonance imaging (MRI), and dual-energy X-ray absorptiometry, which allow for a relatively precise evaluation of muscle CSA and distribution (21–24). Although the use of CT and MRI has increased in veterinary practice, the assessment of muscle mass in clinical settings often relies on general visual inspections and palpation-based evaluations. CT is an imaging modality capable of quantitatively evaluating skeletal muscle CSA and tissue composition. In human medicine, CT is widely used as the gold standard for assessing sarcopenia and muscle mass reduction (21, 25, 26). By distinguishing between muscle and adipose tissue based on Hounsfield unit (HU) values, CT enables the evaluation of not only muscle size but also qualitative characteristics, such as muscle density (21, 22, 25, 26). In veterinary medicine, CT-based studies evaluating skeletal muscles have been increasingly reported, including attempts to quantify muscle mass at specific anatomical levels (27–29). These developments support the use of CT as a reference modality to validate region-specific assessments of the thigh and crus muscles.

Based on these considerations, we hypothesized that thigh musculature would demonstrate significant differences in muscle proportion and CSA at different femoral levels owing to anatomical variations along the femur. We further hypothesized that quantification of the gastrocnemius muscle at a single tibial level would provide a clinically meaningful evaluation of crus muscle mass. In addition, we developed a hindlimb muscle condition score (hMCS), adapted from human hindlimb muscle assessment approaches and specifically designed to evaluate pelvic limb musculature through direct palpation, and anticipated that this score would show measurable associations with CT-derived muscle indices (30). Finally, we hypothesized that pelvic limb muscle mass is associated with biological and clinical variables, including body weight, age, sex, body condition score (BCS), and hMCS.

The objective of this study was to analyze the distribution of pelvic limb musculature according to anatomical location using CT. We aimed to identify optimal femoral measurement levels applicable in clinical practice based on muscle distribution patterns. In addition, we evaluated the clinical validity of the hMCS by examining its association with CT-derived muscle parameters and explored the relationship between pelvic limb muscle mass and individual characteristics.

## 2 Materials and methods

### 2.1 Study population

This prospective observational study was conducted at Kangwon National University Veterinary Teaching Hospital between June and November 2025. All dogs that underwent CT examination during the study period were enrolled, irrespective of the clinical indication for CT. The exclusion criteria were as follows: congenital deformities of the pelvic limb, postoperative bony deformities or structural alterations attributable to prior surgery, and cutaneous or soft-tissue masses involving the pelvic limb that could interfere with the cross-sectional analysis. A total of 41 dogs were enrolled in this study. After applying the exclusion criteria, 39 dogs were included in the final analysis, resulting in 76 pelvic limbs. Baseline data, including identification number, age, sex, neuter status, body weight, and breed, were recorded. The study protocol was approved by the Institutional Animal Care and Use Committee of Kangwon National University (IACUC; KW-250604-2).

### 2.2 Physical examination and hindlimb muscle assessment

**Figure 1:**
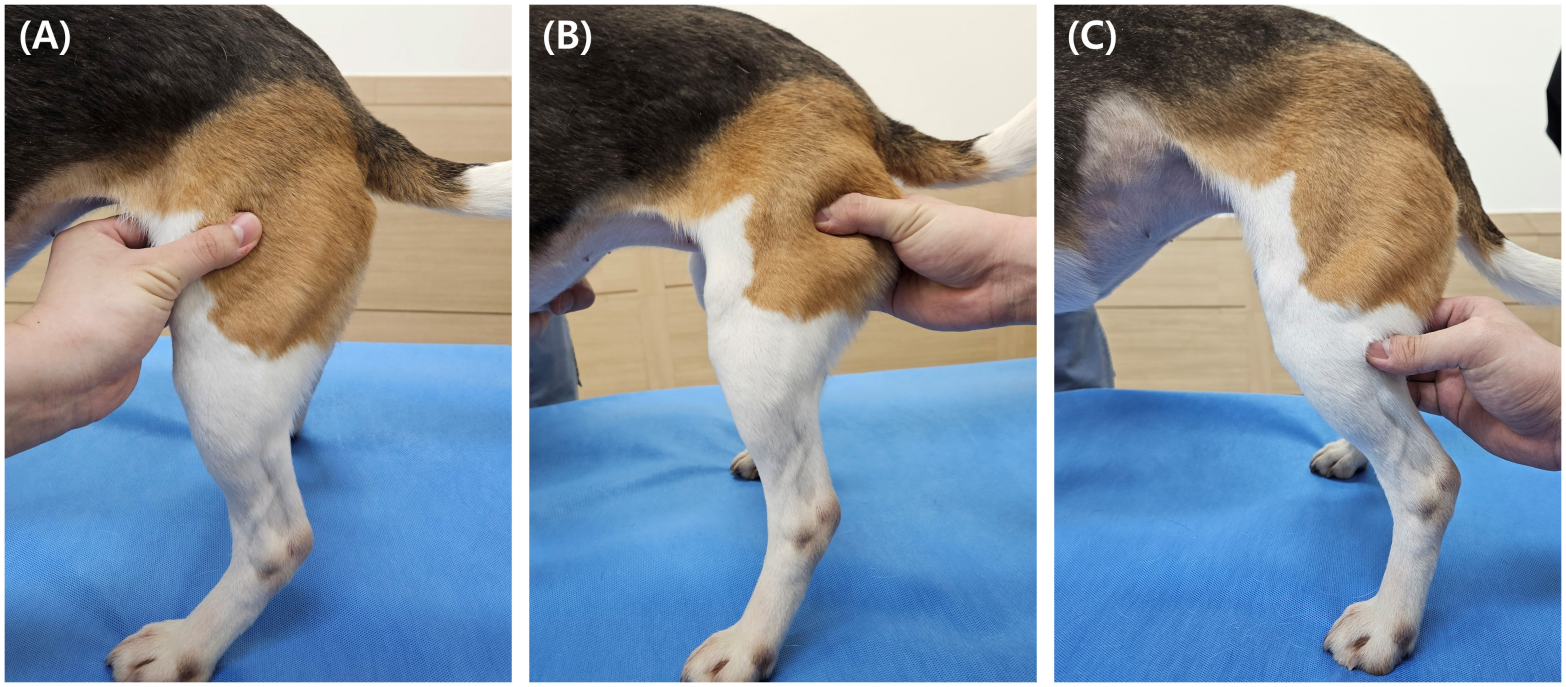
Clinical assessment of hindlimb muscle condition score (hMCS). Evaluation of **(A)** the cranial thigh muscle group, **(B)** the caudal thigh muscle group, and **(C)** the caudal crus muscle group.

BCS was assessed in all dogs using a 9-point scale. In addition, a palpation-based hMCS adapted from hindlimb muscle assessment approaches used in human medicine, has been newly developed and applied (30). The hMCS is listed in Table 1. It was designed to evaluate muscle loss in the pelvic limb in a relaxed standing position (Figure 1). The hMCS was independently assessed by two clinicians experienced in veterinary diagnostic imaging (S.Y. and S.H.), who were blinded to each other's scores and to the CT-based quantitative measurements, although routine clinical information was available during assessment. The thigh was assessed by palpating the cranial (quadriceps femoris) and caudal (hamstrings) muscle groups. In this study, the hamstring muscle group included the biceps femoris, semitendinosus, and semimembranosus muscles. Each muscle group was scored independently on a 4-point scale: 0 (severe atrophy), 1 (moderate atrophy), 2 (mild atrophy), and 3 (normal). The thigh score was calculated by summing the two components, resulting in a score range of 0–6. The crus was assessed by palpating the caudal muscle group, including the gastrocnemius muscle, using the same 4-point scale.

**Table 1:** Hindlimb muscle condition score (hMCS) system.

Score	Femur (anterior)	Femur (posterior)	Tibia (posterior)
3–Normal	Rounded, firm; normal tone; femur not palpable	Full, rounded; normal thickness and tone	Firm muscle belly; normal tone
2–Mild atrophy	Mild flattening; slight bulk reduction; tone preserved	Mild thinning; slight tone reduction	Reduced bulging; tone preserved
1–Moderate atrophy	Reduced bulk; femur palpable	Flattened contour; reduced thickness; femur palpable	Reduced mass; tibia palpable
0–Severe atrophy	Marked atrophy; femur clearly palpable	Minimal to no muscle over femur	Near-complete loss; tibia/fibula palpable

### 2.3 CT acquisition

All CT examinations were performed using a 16-slice CT scanner (Alexion Advance; Canon Medical Systems, Japan). The scanning parameters were set at 120 kVp, 150 mA, and 1-mm slice thickness. All dogs received premedication with butorphanol (0.2 mg/kg) and midazolam (0.2 mg/kg), and anesthesia was induced with propofol (5 mg/kg). Anesthesia was maintained using isoflurane. The dogs were positioned in sternal recumbency, and the stifle joint angle was set to ≥135° using a goniometer to reflect the standing posture. The scanning range included the entire pelvic limb, from the hip to the tarsal joint.

### 2.4 Image analysis

**Figure 2:**
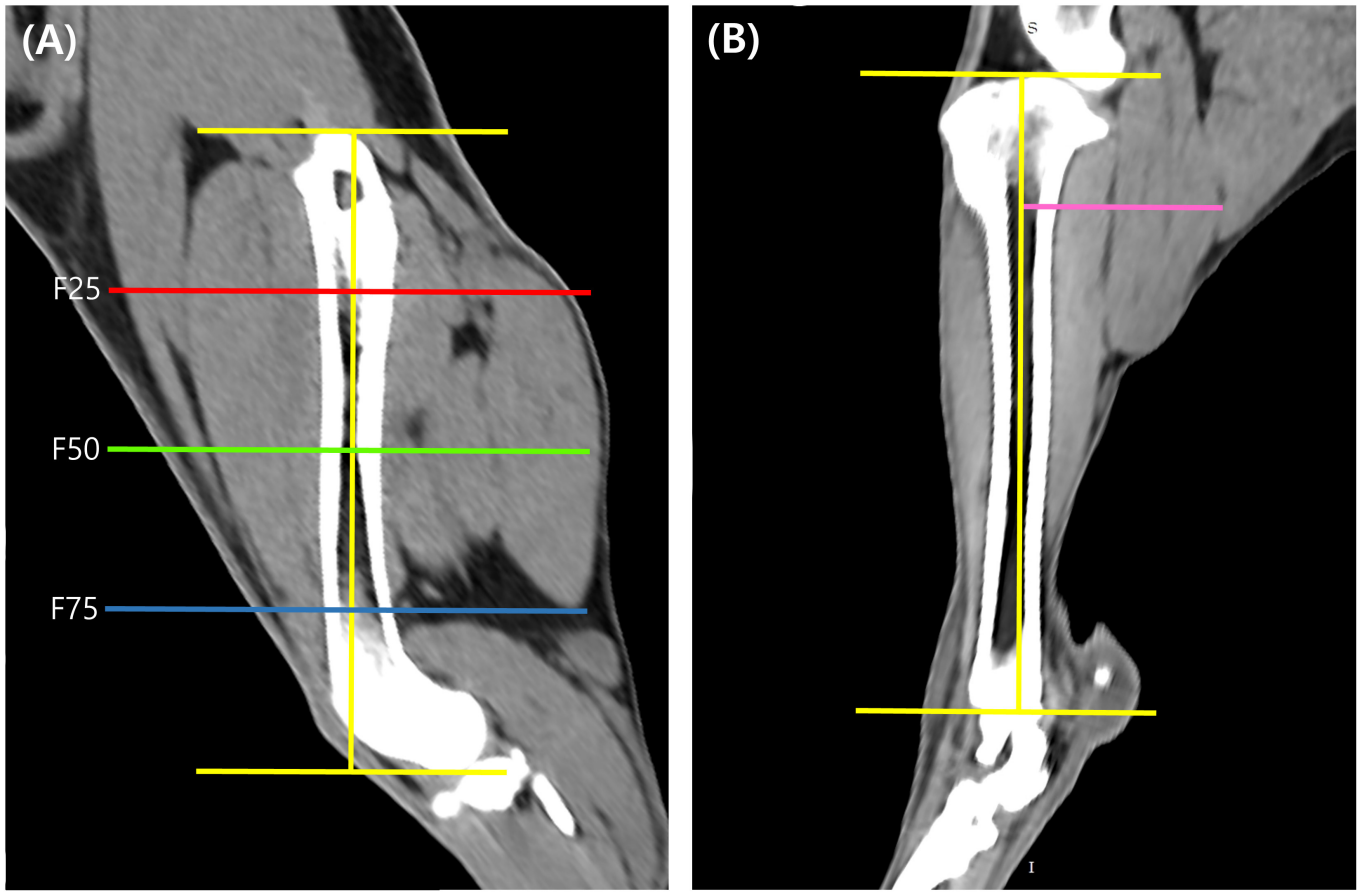
Definition of measurement levels in the femur and tibia on sagittal computed tomography (CT) images. **(A)** Sagittal CT image of the femur demonstrating level selection. The distance from the greater trochanter to the lateral condyle is divided into four equal parts. Cross-sectional levels are defined as F25 (red line), F50 (green line), and F75 (blue line). **(B)** Sagittal CT image of the tibia showing measurement references. The tibial length is defined from the intercondylar eminence to the lateral malleolus. The level of maximal gastrocnemius muscle thickness is indicated (pink line).

**Figure 3:**
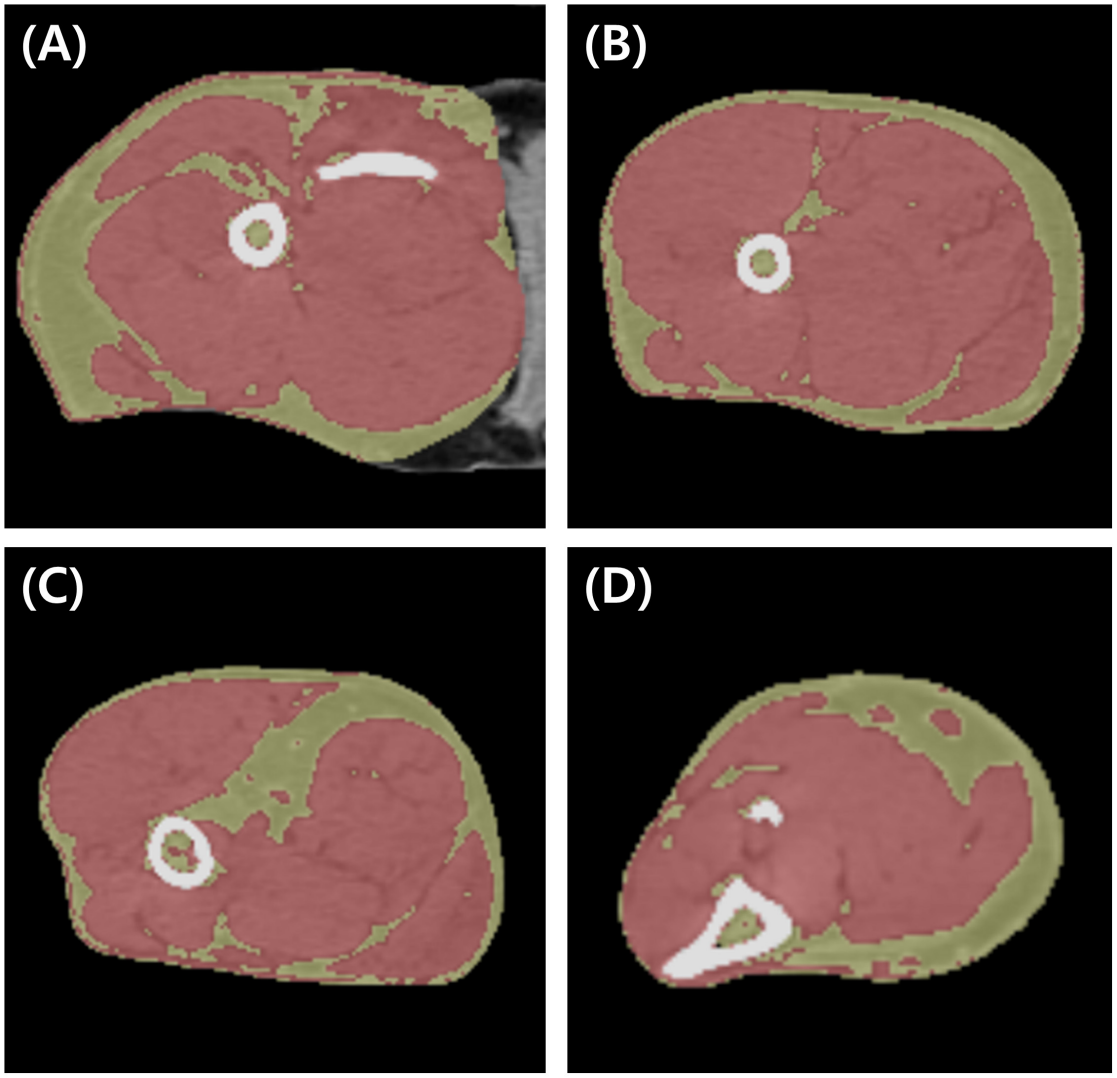
Tissue segmentation based on Hounsfield unit (HU) thresholds in computed tomography (CT) images. **(A–D)** Cross-sectional image at the F25, F50, F75 and the tibia level respectively. Adipose tissue (-190 to−30 HU, yellow color), skeletal muscle (-29 to 150 HU, brown color), and bone (>150 HU, gray color) are shown. Regions within the medullary cavity that were misclassified as muscle or adipose tissue were reassigned to bone.

CT image analysis was performed using Xelis software (Infinitt Healthcare, Korea). Quantitative measurements were performed on transverse CT images using a soft tissue window (window level 60, window width 400). Femoral length was defined as the distance from the greater trochanter to the lateral condyle. Cross-sectional images were obtained at 25%, 50%, and 75% of the femoral length (F25, F50, and F75, respectively; Figure 2A). For tibial analysis, tibial length was defined as the distance from the intercondylar eminence to the lateral malleolus. Unlike the femur, a single tibial level was used because the crus musculature, particularly the gastrocnemius muscle, is predominantly distributed in the proximal tibia, whereas tendinous and ligamentous structures become increasingly prominent distally. On sagittal images, the cross-section demonstrating the maximal thickness of the gastrocnemius muscle was identified and selected as the tibial level (Figure 2B). The tibial location was defined as the ratio of the distance from the intercondylar eminence to this level to the total tibial length. The location of this slice was recorded as a percentage of the total tibial length. At each level, the tissues were segmented using HU thresholds. Adipose tissue was defined as −190 to −30 HU, muscle as −29 to 150 HU, and bone as >150 HU (Figure 3). Regions within the medullary cavity that were misclassified as muscle or adipose tissue were manually reassigned as bone. The CSA (mm^2^) of each tissue component was calculated. Muscle CSA was defined as the absolute muscle area. The muscle ratio was calculated as the muscle CSA divided by the total CSA, where the total CSA was defined as the sum of the muscle, fat, and bone CSA. In addition, CT-based limb circumference (CTC) was measured at each level and defined as the perimeter of the external limb boundary on transverse CT images. All imaging analyses were performed by a single investigator.

### 2.5 Statistical analysis

All statistical analyses were performed using SPSS version 29.0 (IBM Corp., Armonk, NY, USA). Normality of continuous variables was assessed using the Shapiro–Wilk test, and descriptive statistics were reported. Because both pelvic limbs of each dog were included, a repeated-measures structure was considered in the analysis. Differences in muscle CSA and muscle ratio among femoral levels (F25, F50, and F75) were analyzed using linear mixed models (LMM), with the level included as a fixed effect and individual dogs included as a random effect. To evaluate whether a single femoral level could represent the overall femoral muscle mass, the correlations of muscle CSA between F50 and other femoral levels (F25 and F75) were assessed using Spearman's correlation analysis. *Post-hoc* comparisons were performed using Bonferroni correction when appropriate. Associations between muscle CSA or muscle ratio and clinical variables were analyzed using LMM, with the following variables included as fixed effects: body weight, age, sex, BCS, and hMCS. Interobserver reliability of the hMCS was evaluated using the intraclass correlation coefficient (ICC). The relationship between CTC and muscle CSA was assessed using Spearman correlation analysis. For all Spearman correlation analyses, each limb was treated as a separate observation because the objective was to evaluate measurement-level associations rather than dog-level effects. Statistical significance was set at *p* < 0.05.

## 3 Results

### 3.1 Study population and baseline characteristics

A total of 39 dogs (76 hindlimbs) were included in the final analysis. The baseline characteristics of the study population, including breed, body weight, age, sex distribution, BCS, and hMCS, are summarized in Table 2. Based on the assessment of normality, body weight, age, BCS, and hMCS are presented as median (interquartile range) and sex as frequencies for castrated males, intact males, spayed females, and intact females.

**Table 2:** Baseline characteristics of the study population (*n* = 39 dogs; 76 limbs).

Variable	Value	Score (*n*)
Breed (*n*)	Beagle (13), Bichon frise (7), Mixed (3), Miniature poodle (3), Chihuahua (2), Maltese (2), Shih-tzu (2), Cocker spaniel (1), Dachshund (1), German shepherd (1), Great pyrenees (1), Jindo (1), Sapsali (1), Yorkshire terrier (1)	
Weight (kg)^a^	8.0 (4.6–9.1)	
Age (years)^a^	6.5 (1.0–11.8)	
Sex (*n*)	CM (11), IM (13), SF (13), IF (2)	
BCS (1-9)^a^	4 (3-6)	1 (0), 2 (4), 3 (9), 4 (13), 5 (3), 6 (6), 7 (2), 8 (1), 9 (1)
hMCS, Femur (0–6)^a^	6 (4-6)	0 (0), 1 (4), 2 (3), 3 (4), 4 (13), 5 (12), 6 (20)
hMCS, Tibia (0–3)^a^	2 (2-3)	0 (2), 1 (6), 2 (31), 3 (37)

^a^Values are presented as median (interquartile range) CM, castrated male; IM, intact male; SF, spayed female; IF, intact female; BCS, body condition score; hMCS, hindlimb muscle condition score.

### 3.2 CT-derived muscle measurements by level

#### 3.2.1 Femoral muscle CSA and muscle ratio across levels

**Figure 4:**
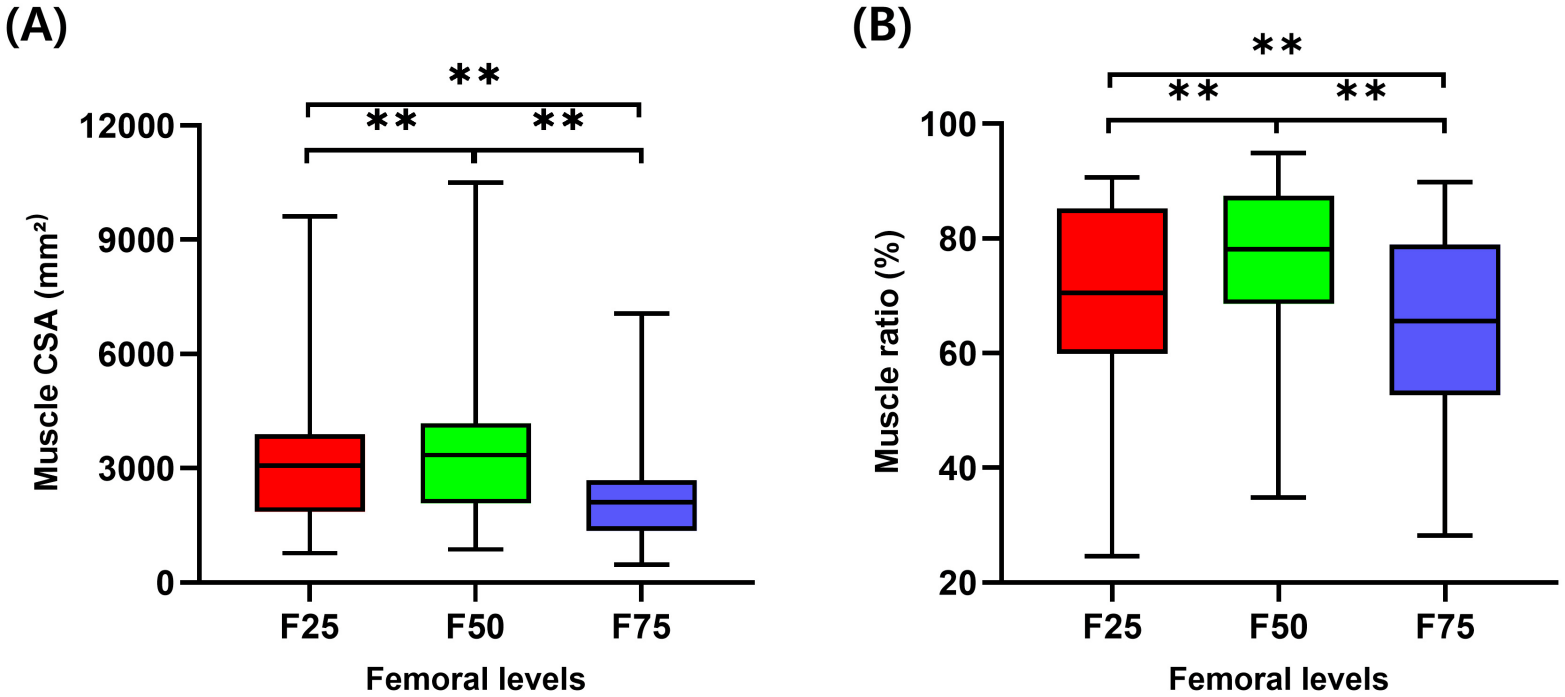
Distribution of muscle cross-sectional area (CSA) and muscle ratio across femoral levels. Box plots showing **(A)** muscle CSA and **(B)** muscle ratio at F25, F50, and F75. Boxes represent the interquartile range, the center line indicates the median, and whiskers represent the minimum and maximum values. Significant differences between groups are determined using a linear mixed model (**p < 0.01).

Descriptive statistics for muscle CSA, total CSA, and muscle ratio at the three femoral levels (F25, F50, and F75) are presented in Table 3. Muscle CSA differed significantly across the levels (LMM, *p* < 0.001; Figure 4A). The mean muscle CSA was highest at F50, followed by F25 and F75. In the *post hoc* comparisons, both F50 and F25 were significantly higher than F75 (*p* < 0.001). The muscle ratio also differed significantly by level (LMM, *p* < 0.001; Figure 4B), with the highest ratio at F50, followed by F25, and the lowest at F75. The *post hoc* analysis indicated that the differences among all levels were significant (all *p* < 0.001). Muscle CSA at F50 showed strong positive correlations with those at F25 (*r* = 0.936) and F75 (*r* = 0.982).

**Table 3:** CT-derived muscle cross-sectional area (CSA) and muscle ratio at femoral and tibial levels.

Level	Muscle CSA (mm^2^)	Total CSA (mm^2^)	Muscle ratio (%)	Tibial location (%)
F25	3,063 (1,862–3,890)	4,350 (2,987–5,676)	70.6 (59.9–85.3)	
F50	3,342 (2,083–4,183)	4,517 (3,159–5,301)	78.1 (68.6–87.3)	
F75	2,108 (1,353–2,678)	3,258 (2,628–3,951)	65.3 (52.7–78.7)	
Tibia	1,223 (792–1,492)	1,949 (1,479–2,353)	64.3 (56.0–74.5)	11.9 (9.9–15.0)

All values are presented as median (interquartile range). CSA, cross-sectional area.

#### 3.2.2 Tibial muscle CSA and muscle ratio

Descriptive statistics for the muscle CSA, total CSA, and muscle ratio of the tibia are presented in Table 3. Muscle CSA and ratio were evaluated at a single level corresponding to the maximal CSA of the gastrocnemius muscle. The tibial location, defined as the level of maximal gastrocnemius muscle CSA, corresponded to 11.9% (9.9–15.1) of the total tibial length. At this level, the median muscle CSA was 1,223 mm^2^ (792–1,492), and the median muscle ratio was 64.25% (56.0–74.5).

### 3.3 LMM analysis of factors associated with muscle CSA and muscle ratio

#### 3.3.1 Factors associated with muscle CSA

The results of the LMM analyses with muscle CSA as the dependent variable at each femoral (F25, F50, and F75) and tibial level are presented in Table 4. Body weight was significantly and positively associated with muscle CSA at all levels (*p* < 0.001). Age, sex, and BCS were not significantly associated with muscle CSA at any level. hMCS was positively associated with muscle CSA at F50, F75, and the tibial level (*p* < 0.01), but not at F25 (*p* = 0.298).

**Table 4:** Linear mixed model (LMM) analysis for factors associated with muscle cross-sectional area (CSA) and muscle ratio.

Outcome	Predictor	Estimated coefficients [*β* (95% CI)]			
		F25	F50	F75	Tibia
Muscle CSA	Weight	225.3^**^ (148.7–302.0)	206.3^**^ (124.9–287.7)	78.3^**^ (34.2–122.5)	63.1^**^ (47.5–78.6)
	Age	−62.8 (-135.9–10.2)	−64.8 (-157.9–28.4)	−47.2 (-117.0–22.6)	−16.1 (-43.2–11.0)
	BCS	−82.4 (-276.0–111.1)	−183.3 (-445.0–78.4)	39.0 (-167.8–245.8)	26.4 (-44.4–97.3)
	hMCS	51.6 (-46.7–150.0)	143.0^**^ (51.7–234.2)	167.1^**^ (74.9–259.3)	199.9^**^ (109.5–290.3)
Muscle ratio	Weight	0.275 (-0.307–0.857)	−0.054 (-0.606–0.498)	0.334 (-0.276–0.943)	−0.096 (-0.759–0.567)
	Age	−0.859^*^ (-1.563–−0.155)	−0.795^*^ (-1.492–−0.099)	−0.775 (-1.589–−0.039)	−0.846^*^ (-1.680–−0.013)
	BCS	−7.912^**^ (-10.205–−5.620)	−6.368^**^ (-8.740–−3.995)	−7.172^**^ (-9.390–−4.954)	−7.229^**^ (-9.674–−4.784)
	hMCS	2.225^**^ (0.956–3.494)	2.511^**^ (1.326–3.696)	3.188^**^ (1.391–4.986)	5.717^**^ (2.241–9.194)

^*^P < 0.05; ^**^P < 0.01 CSA, cross-sectional area; CI, confidence intervals; BCS, body condition score; hMCS, hindlimb muscle condition score.

#### 3.3.2 Factors associated with muscle ratio

The results of the LMM analyses with muscle ratio as the dependent variable at each femoral (F25, F50, and F75) and tibial level are presented in Table 4. Body weight was not significantly associated with the muscle ratio at any level. Age showed a significant negative association with the muscle ratio at F25, F50, and tibial level (*p* < 0.05), but not at F75. Sex showed a significant association at F25, F75, and tibial level (*p* = 0.002), but not at F50. Both BCS and hMCS were significantly associated with the muscle ratio at all levels (all *p* < 0.001), with BCS showing a negative association and hMCS showing a positive association.

### 3.4 Interobserver reliability of hMCS

The interobserver reliability of the hMCS was high for both the femoral and tibial assessments. The ICCs for the femur and tibia were 0.978 (95% CI, 0.966–0.986) and 0.960 (95% CI, 0.934–0.975), respectively.

### 3.5 Correlation between CTC and muscle CSA

The relationship between CTC and muscle CSA was evaluated using Spearman's correlation analysis at each femoral (F25, F50, and F75) and tibial level. Significant positive correlations were observed at all levels (*p* < 0.01), with correlation coefficients ranging from 0.681 to 0.729, indicating moderate to strong associations.

## 4 Discussion

This study used CT-based analysis to characterize hindlimb muscle distribution, identify optimal measurement levels, and evaluate the clinical relevance of hMCS. As hypothesized, muscle CSA and muscle ratio differed among the femoral levels, with F50 showing the highest values, supporting its use as a representative level. In the tibia, the maximal CSA of the gastrocnemius muscle was consistently located within the proximal region, supporting its clinical applicability for assessing crus musculature in small animal practice. Muscle indices were associated with body weight, age, sex, BCS, and hMCS. Notably, hMCS showed excellent interobserver reliability and significant associations with CT-derived muscle indices, supporting its potential use as a complementary clinical tool for hindlimb muscle assessment.

In this study, the femoral measurement levels were defined at 25%, 50%, and 75% of the total femoral length. This proportional approach was adopted to exclude joint-adjacent regions while uniformly capturing changes in muscle distribution along the longitudinal axis of the femur. The proximal femur includes complex soft tissue structures and muscle origins around the hip joint, whereas the distal femur includes structures adjacent to the stifle joint, where the relative proportions of tendons and bone increase (18). Dividing the femoral length into proportional segments representing the proximal, mid, and distal regions is considered a more rational approach in terms of reproducibility and clinical applicability. Unlike the femur, a single level was defined for the tibia. This approach reflects the anatomical characteristics of the gastrocnemius muscle, which courses along the caudal aspect of the tibia and has a relatively consistent morphological distribution (18). Rather than establishing multiple tibial levels, a single level based on the maximal CSA was selected to provide a simplified yet functionally relevant assessment framework.

Significant differences in muscle CSA and muscle ratio were observed among F25, F50, and F75. These findings highlight the importance of measurement standardization in hindlimb muscle assessments, as variations in measurement levels may lead to apparent differences in muscle mass that reflect positional variations rather than true physiological changes. F50 showed the highest values for both muscle CSA and muscle ratio. This finding likely reflects the anatomical concentration of major propulsive muscle groups within the mid-femoral region, where the muscle bulk is most prominently developed (18). This observation is also consistent with previous thigh girth measurements demonstrating greater values at the 50% femoral level than at more distal locations (16). In contrast, the proximal level (F25) may be influenced by the adjacent hip joint structures and heterogeneous soft tissue components, whereas the distal level (F75), approaching the stifle joint, contains increasing proportions of bone and tendinous tissue, which may reduce the relative contribution of muscles within the CSA (18). Muscle CSA at F50 was also strongly correlated with that at F25 and F75, indicating that measurements obtained at this level closely reflect muscle mass across the femur. Therefore, considering its maximal muscle representation, anatomical suitability, and strong correlation with other femoral levels, F50 appears to be an optimal measurement level for femoral muscle evaluation.

In a previous ultrasonographic study evaluating canine femoral musculature, the quadriceps femoris demonstrated the greatest CSA in the proximal to mid-femoral region, whereas the hamstring muscle group showed the largest CSA in the mid-femoral region (17). These findings are consistent with the results of the present study, in which the mid-femoral level (F50) exhibited the highest muscle CSA, followed by the proximal level (F25). This agreement across different imaging modalities supports the concept that the mid-femoral region represents the area of maximal muscle development and may serve as a reliable site for muscle assessment. This characteristic may also be applicable to simple morphometric measurements in clinical practice, such as limb circumference using a measuring tape, suggesting that the mid-femoral level could be utilized as a standardized measurement site.

In tibial analysis, previous studies evaluating tibial muscle mass in dogs have reported that measurements are often performed at the proximal segments of the tibia (31, 32). In the present study, the maximal cross-sectional location of the gastrocnemius muscle was distributed within a relatively narrow proximal region corresponding to approximately 12% of the total tibial length, supporting the measurement locations reported in previous studies. This finding reflects the anatomical development of the gastrocnemius muscle, which is most prominently formed in the proximal tibia, and suggests that this region may serve as an appropriate cross-section for tibial muscle mass assessment (18). The present results further indicate that a single-level assessment based on the maximal gastrocnemius cross-section may provide a simplified and reproducible approach for tibial muscle evaluation. This approach may be particularly useful in clinical settings that require repeated follow-ups or when time and resources are limited.

Body weight demonstrated a strong positive association with muscle CSA across all levels, indicating that the absolute muscle area largely reflects overall body size. This finding is consistent with that of a previous CT-based study (33). In contrast, body weight was not significantly associated with the muscle ratio, suggesting that the relative muscle composition is independent of body size. BCS did not show a consistent association with muscle CSA but demonstrated a clear negative association with muscle ratio at all evaluated levels. For each one-point increase in BCS, the muscle ratio decreased by 6–8%. This pattern suggests that rather than an actual decrease in muscle mass, increased adiposity leads to a reduction in the relative proportion of muscle within a given CSA. These findings suggest that muscle ratio may provide a more body size–independent indicator of muscle status than absolute muscle CSA, which was significantly associated with body weight.

A previous study reported that obesity associated with aging can induce insulin resistance and contribute to the progression of sarcopenia (34). However, in the present study, BCS was not significantly associated with muscle CSA. This may be attributed to the use of BCS as a surrogate marker of adiposity and the inclusion of a heterogeneous population with a wide age range. BCS primarily reflects adiposity rather than muscle mass (13, 14). Therefore, the assessment of muscle conditions may require additional evaluation methods that directly quantify muscle status.

Aging is associated with compositional changes in the skeletal muscles, including increased fat accumulation and a relative decline in muscle proportion (35). Similar age-related alterations in body composition have been reported in dogs (27, 36, 37). In the present study, muscle CSA showed a decreasing trend with increasing age; however, this trend did not reach statistical significance across all evaluated levels. In contrast, the muscle ratio demonstrated a consistent negative association across multiple levels, decreasing by approximately 0.8% per year. These findings suggest that aging in dogs may have a greater impact on muscle composition than on absolute muscle size, leading to a reduction in the muscle ratio. Therefore, evaluating compositional indices, such as the muscle ratio alongside absolute muscle area, may provide a more sensitive approach for detecting early age-related changes in muscle status.

Sex hormones, such as testosterone and estrogen, play a key role in the regulation of skeletal muscle mass and fat distribution. Neutering has been associated with alterations in body composition, as a reduction in sex hormones may decrease the basal metabolic rate and increase appetite, leading to increased adiposity and changes in lean tissue proportion (38–42). In the present study, sex was not significantly associated with absolute muscle CSA but showed level-dependent differences in the muscle ratio. At the proximal and distal femoral levels, intact males exhibited approximately 11–14% higher muscle ratios than spayed females, whereas in the tibia, castrated males showed approximately 14% higher values. No significant differences were observed at the mid-femoral level. Although these findings may suggest a potential influence of sex and neuter status on muscle composition, they should be interpreted with caution because of the limited sample size within several subgroups, particularly intact females. Likewise, the absence of significant associations should not be interpreted as evidence that sex or neuter status has no effect on muscle parameters. Furthermore, the inconsistent direction of the observed differences across measurement levels and the use of selected cross-sectional levels that may not fully reflect overall hindlimb muscle volume should also be considered.

In human medicine, the importance of physical examinations for assessing muscle and fat loss across various body regions, including the limbs, has been emphasized in the diagnostic criteria for malnutrition (30). In contrast, conventional MCS system in veterinary medicine has primarily been designed to evaluate overall muscle status based on epaxial musculature, limiting its ability to assess regional muscle changes (13, 14). Based on this concept, the present study developed a novel palpation-based scoring system, hMCS, that specifically targets the thigh and crus muscles. hMCS demonstrated consistent positive associations with both muscle CSA and ratio. In particular, at the mid- and distal femoral levels as well as in the tibia, each one-point increase in hMCS was associated with an increase in muscle CSA of approximately 140–200 mm^2^, whereas the muscle ratio increased by approximately 2–6%, depending on the level. These findings indicate that hMCS reflects CT-derived quantitative muscle indices. Particularly, the consistent association between hMCS and muscle ratio provides additional support for the use of hMCS, as muscle ratio was less affected by body size than absolute muscle CSA. In addition, hMCS demonstrated excellent interobserver reliability, indicating high reproducibility.

Previous studies have reported that body condition score and muscle condition score assessments may vary among evaluators with different levels of training and experience, highlighting the importance of standardized assessment guidelines and regular training (43). In addition, repeatability and reproducibility may vary according to MCS category (44). The limited number of dogs with moderate and severe muscle atrophy in the present study may restrict interpretation of hMCS performance in these categories. Future studies including a broader distribution of hMCS scores, particularly dogs with moderate and severe muscle atrophy, are warranted to further validate the applicability of hMCS across diverse clinical populations.

Taken together, these findings suggest that hMCS is a non-invasive, cost-effective, simple, and rapid clinical tool for assessing muscle status, particularly in settings where access to CT is limited. In clinical practice, hMCS may serve as a complementary tool for monitoring longitudinal changes in hindlimb muscle mass during recovery following orthopedic surgery, throughout rehabilitation programs, and in patients with chronic diseases and age-related sarcopenia.

CTC demonstrated moderate to strong positive correlations with muscle CSA at all evaluated levels. These findings suggest that the cross-sectional circumference measured at a given level reasonably reflects the absolute muscle mass at that site. However, because CTC includes not only muscle but also subcutaneous fat and other soft tissues within the cross-section, it does not directly reflect relative muscle composition or body composition changes. Therefore, caution is required when interpreting CTC as a measure of muscle mass. Accordingly, CTC should be interpreted as an indirect indicator of absolute muscle mass rather than a measure of muscle composition.

The primary limitation of this study is the heterogeneous study population, as dogs were enrolled irrespective of the indication for CT examination. Underlying diseases, activity levels, nutritional status, and overall clinical history may have influenced muscle mass and body composition and contributed to the observed associations between muscle indices and individual variables. Additionally, the relatively modest sample size, single-center design, and predominance of small-breed dogs with limited representation of different breed and conformation types may limit the generalizability of the findings. Therefore, the applicability of the proposed measurement levels and hMCS to dogs with markedly different body conformations remains uncertain. Despite these limitations, consistent level-dependent differences in muscle distribution and significant associations between muscle parameters and individual factors, such as body weight, age, sex, BCS, and hMCS were identified, supporting the anatomical and clinical relevance of the proposed measurement approach. Although CT-derived quantitative measurements were obtained using a standardized threshold-based segmentation protocol, formal assessment of intraobserver reproducibility was not performed and may warrant further investigation. Future studies involving larger, breed- and conformation-diverse, and disease-specific populations may further refine the clinical interpretation of hindlimb muscle assessment indices.

In conclusion, this study characterized the distribution of hindlimb skeletal muscles in dogs using CT-based quantitative analysis and identified anatomically optimal measurement levels. The mid-femoral level (F50) was the most appropriate site for representing femoral muscle mass, whereas a single-level assessment based on the maximal CSA of the gastrocnemius muscle provided a practical approach for the tibia. Palpation-based hMCS showed significant associations with CT-derived muscle indices and excellent interobserver reliability, supporting its clinical applicability. Hindlimb muscle parameters were also associated with body weight, age, sex, and BCS, indicating a close relationship between regional muscle assessment and overall body composition. These findings support the standardization of imaging-based hindlimb muscle evaluation and the integration of clinically applicable muscle assessment tools into routine practice.

## Data Availability

The original contributions presented in the study are included in the article/supplementary material, further inquiries can be directed to the corresponding author.
